# Contribution of cell proliferation to axial elongation in the red flour beetle *Tribolium castaneum*

**DOI:** 10.1371/journal.pone.0186159

**Published:** 2017-10-09

**Authors:** Rodrigo E. Cepeda, Renato V. Pardo, Constanza C. Macaya, Andres F. Sarrazin

**Affiliations:** Instituto de Química, Pontificia Universidad Católica de Valparaíso, Valparaíso, Chile; University of Otago, NEW ZEALAND

## Abstract

Most arthropods generate their posterior bodies by adding segments periodically, as the embryo grows, from a posteriorly located region called the segment addition zone. This mode of segmentation is shared with vertebrates and relies on oscillatory mechanisms, where the temporal periodicity of a clock is translated into repetitive spatial patterns. This ordered anterior-to-posterior pattern is achieved at the same time as the tissue elongates, opening the question of the functional coordination between the mechanisms of segmental patterning and posterior growth. The study of these processes in different arthropods has played an important role in unravelling some of the molecular mechanisms of segment formation. However, the behavior of cells during elongation and how cellular processes affect this segmental patterning has been poorly studied. Cell proliferation together with cell rearrangements are presumed to be the major forces driving axis elongation in the red flour beetle *Tribolium castaneum*. However, there still no strong evidence about the role and distribution of cell proliferation within the embryo. In this study, we propose to address these questions by using whole embryo cultures and pharmacological manipulation. We show that considerable cell proliferation occurs during germband elongation, measured by incorporation of the nucleoside analog of thymidine 5-Ethynyl-2’-deoxyuridine, EdU. Moreover, proliferating cells appeared to be spread along the elongating embryo with a posterior bias at early segmentation. In addition, when we blocked cell division, treated germbands were always shorter than controls and in some cases not able to fully elongate, even when control embryos already started to retract and leg buds are evident. Finally, we found that the absence of cell proliferation has no apparent effect on segmental patterning, as evidenced by *Tc-engrailed* (*Tc-en*) gene expression.

## Introduction

A segmented body plan is one of the main, and presumably ancestral, characteristics of arthropods. Given the modular nature of each segment, segmented organisms have high flexibility for adaptation, which has been considered as a very advantageous way of promoting variation, making them the most diverse group of animals on the planet [1, 2]. In the fruit fly *Drosophila melanogaster*, all body segments are formed by a nearly simultaneous subdivision of the blastoderm. This segmental pattern is established by a well-defined genetic cascade that operates in a syncytium, where maternal factors diffuse and trigger the consecutive activation (or repression) of the zygotic gap, pair-rule and segment-polarity genes [3, 4]. Later, during gastrulation, *Drosophila* germband extends along the anteroposterior (AP) body axis doubling its length while simultaneously narrowing in width, in a process driven mainly by cell intercalation and oriented cell divisions [5, 6]. In contrast to *Drosophila*, in the vast majority of arthropods (Chelicerata, Myriapoda, Crustacea and short germ insects), most segments arise sequentially from an apparently undifferentiated region located at the caudal end of the embryo, called the segment addition zone (SAZ) [7]. Interestingly, this mode of segmentation is also found in vertebrates [8] and relies in both cases on oscillatory mechanisms, where the temporal periodicity of a clock is translated into repetitive spatial patterns [9–13]. This ordered anterior-to-posterior pattern is achieved at the same time as the tissue elongates, opening the question of the functional relevance of one process (elongation) on the other (segmentation), and vice versa.The red flour beetle *Tribolium castaneum* has become a prominent comparative model organism, being the second best studied arthropod after *Drosophila melanogaster* [14–16]. Within the last years, diverse genetic and molecular tools, as well as imaging and embryonic techniques, have been developed in *Tribolium* [12, 17–20], providing the opportunity to understand a wide range of developmental processes and certainly to give insights into the morphological evolution of arthropods. During *Tribolium* development, embryonic extension along the AP axis involves considerable cell and tissue remodeling, where a short and wide germ anlage turns into a long and narrow germband. This dramatic change in embryo morphology must be the result of the combination of several types of cellular processes controlling cell number, size, shape and position in relation to their neighbors. Given that sequentially segmenting arthropods (like *Tribolium*) generate their posterior segments from a relatively small region at the end of the embryo (SAZ), one can expect that cell proliferation is the main driving force for germband elongation [4]. However, the relative contribution of this and other cellular behaviors during axial elongation and segmentation has been neglected until very recently.

Recent work on the milkweed bug *Oncopeltus fasciatus* and *Tribolium* [12, 20–22], as well as few studies on crustaceans [23, 24], have contributed to unravel the role that cell division may play in sequential segmentation. Auman et al. [22], based on cell division profiles and gene expression patterns in *Oncopeltus*, proposed a model for segmentation where they defined three embryonic domains: A posterior SAZ containing undifferentiated cells with relatively high level of proliferation; an anterior SAZ with cells undergoing segmental specification and low levels of cell division; and the posterior part of the segmented germband, formed by proliferating cells. Similar results, using EdU incorporation, were found in the crustacean *Thamnocephalus*, in which another interesting findings were a variable number of proliferating cells present in the SAZ throughout axial elongation and that 85–90% of those cell divisions were oriented along the AP axis [24]. In *Tribolium*, not such an obvious orientation was found when mitotic cells stained with TOPRO-3 iodide were analyzed, but a peak of proliferation during the transition from mid- to late-germband elongation [12]. On the other hand, Nakamoto et al. [21] mathematically modelled posterior segmentation in the absence of cell division and concluded that elongation in *Tribolium* can occur without posteriorly localized cell proliferation. Nevertheless, when they compared the changes in the elongating germband area at different stages, they found an increase in area of about 41% throughout elongation and they estimated that the original SAZ needed to be about 18% larger to account for the final area. This suggests that some of the driving force for axial elongation may come from cell divisions and that cell rearrangements alone cannot account for the whole process. For example, a new described family of Toll genes required for convergent extension movements in *Drosophila* [25], when it was analyzed functionally, was found to be necessary for cell intercalation in *Tribolium*, but only partially affecting axial elongation without disrupting segment specification [26]. Interestingly, in the same study they found that eliminating Toll function in the spider *Parasteatoda* caused wider germbands without affecting their length [26].

We decided to evaluate the role of cell proliferation during axial elongation by using whole embryo cultures and pharmacological manipulation [12, 19]. We found considerable cell proliferation during germband elongation, measured by incorporation of the nucleoside analog of thymidine 5-Ethynyl-2’-deoxyuridine, EdU. Moreover, proliferating cells appeared to be spread along the elongating embryo with a posterior bias. In addition, when cell proliferation was inhibited in early- and mid-elongated stages, as well as by pharmacological injection, treated germbands were always shorter than controls and in some severe cases, posteriorly truncated. Finally, we found that the absence of cell division had no apparent effect on segment patterning, as was evidenced by *Tc-engrailed* (*Tc-en*) expression.

## Materials and methods

Beetle husbandry, whole embryo cultures, EdU labeling, drug treatments, embryonic viability assay by propidium iodide incubation, single-embryo *in situ* hybridization and embryo length estimation were carried out as described in Macaya et al. [19]. A *Tc-engrailed* digoxigenin-labeled RNA probe was synthesized from a PCR amplified template sequence (forward primer 5′-TGCAAGTGGCTGAGTGT-3′ and reverse primer 5′-GCAACTACGAGATTTGCCTTC-3′) cloned into pGEM-T. For chemical DNA synthesis inhibition, we used a mix of aphidicolin (APH; Sigma A0781) and hydroxyurea (HU; Sigma H8627). A working solution of 150 μM APH (stock solution 10 mg/mL in DMSO) and 20 mM HU was freshly prepared in insect medium for each experiment. We found in literature that aphidicolin was used in Drosophila cell culture (25 μM APH for 1 week) [27] and injected, combined with hydroxyurea, in Drosophila syncytial blastoderm embryos (300 μM APH and 10 mM HU) [28]. Given that neither APH nor HU have been used before in whole-embryo insect culturing, we used the most common concentration found in the literature for zebrafish embryo culture [29, 30]. Moreover, the effect of APH+HU on cell proliferation at the selected concentration was tested on dissected germbands also exposed to 200 μM EdU. EdU staining was absent in embryos treated with APH+HU (S1 Fig). Additionally, we checked embryo viability during drug exposure, by propidium iodide (PI) incubation [19]. APH+HU-treated embryos showed few cells labeled with PI, similar to controls (S1 Fig). For injection, embryos at early elongation (t = 0h; horseshoe stage) were selected and aligned on a Petri dish covered with wet filter paper and a mesh. Injection was performed with 150 μM APH and 20 mM HU or DMSO (25%) on the opposite side where the germband is in the egg. After injection, embryos were reared at 30°C and photographed at 8 and 24 hours post injection using a Leica MZ10F fluorescence stereoscope with a Leica DFC 450 CCD camera.

### Fluorescence intensity quantification

After fixation in 4% formaldehyde, germbands were flat mounted with ProLong Gold mounting medium with DAPI (Molecular Probes P36935) and photographed (10x objective) using a Nikon Eclipse Ci epi-fluorescence microscope with a Nikon DS-Fi2 CCD camera. The same camera acquisition settings were used for all EdU images (RGB color type and TIFF format images; exposure time was 800 ms with no gain modulation). The fluorescence intensities of EdU-labeled embryos were measured using ImageJ. We split color channels keeping the red channel. The region of interest (SAZ or trunk, see below) was manually demarcated using the Segmented Line tool and the measurement parameters (area, integrated density and the mean grey value) were selected and registered. Background fluorescence intensity (FI) was also quantified, measuring the same area near the embryo and subtracted from the FI of the same embryo. This procedure was carried out with each embryo analyzed. The final FI value obtained represents the amount of fluorescence per area (FI/μm^2^). We also determined the number of cells that incorpored EdU related to the germband area analyzed (S2, S3 and S4 Figs). Using the ImageJ options *Image > Adjust > Threshold* and *Process > Binary > Convert to Mask* we converted the images to black and white based on the calculated threshold that was adjusted to 2%– 3.8%. Then we used *Process > Binary > Watershed* to establish the cell borders. Finally, by using the option *Analyze > Analyze Particles* we determined the number of labeled cells, considering particle size range between 42.2 and infinite (pixels^2^) and *Circularity* values between 0.2 and 1.0 (we also selected the option to show outlines). In order to determine the border between the SAZ and the trunk of the embryo, we used *Tc-caudal* as a marker of the SAZ, given that the expression of this gene becomes restricted to this region during the entire germband elongation [31]. We measured the length of the SAZ covered by the expression of *Tc-caudal* along the germband (*n* = 5 for early-elongated embryos; *n* = 6 for mid-elongated embryos) and we estimated the percentage of SAZ length along the entire germband (S5 Fig).

### Statistical analysis

Data were analyzed using the Graphpad Prism V6.1 statistical software. Practically all data showed a normal distribution (Shapiro-Wilk test; α = 0.05). Paired t-test was used to determine the statistical significance of our experiments (*P<0*.*05*). To determine statistical differences between non-normally distributed data, we used Wilcoxon’s paired test (*P<0*.*05*). For EdU labeling, we used *n* = 12 embryos at early elongation and *n* = 10 embryos at mid elongation stages. For APH+HU treatments at early elongation stages, we used *n* = 10 embryos (2 h treatment) and *n* = 11 embryos (3 h treatment) for sham treatment controls; *n* = 14 embryos (2 h treatment) and *n* = 10 embryos (3 h treatment) for drug-treatments. At mid-elongation stages, *n* = 11 embryos for all experiments. *Tc-en in situ* hybridization was performed and analyzed in *n* = 10 embryos for t = 0h (control); *n* = 9 embryos (t = 1h) and *n* = 7 embryos (t = 2, 3, 4h) for control and drug treatments. Segment length was analyzed in *n* = 5 embryos in all cases, except for APH+HU treatment (t = 2h), *n* = 6. Data in the text are shown as mean ± standard deviation.

## Results and discussion

### Considerable cell proliferation during germband elongation

**Fig 1:**
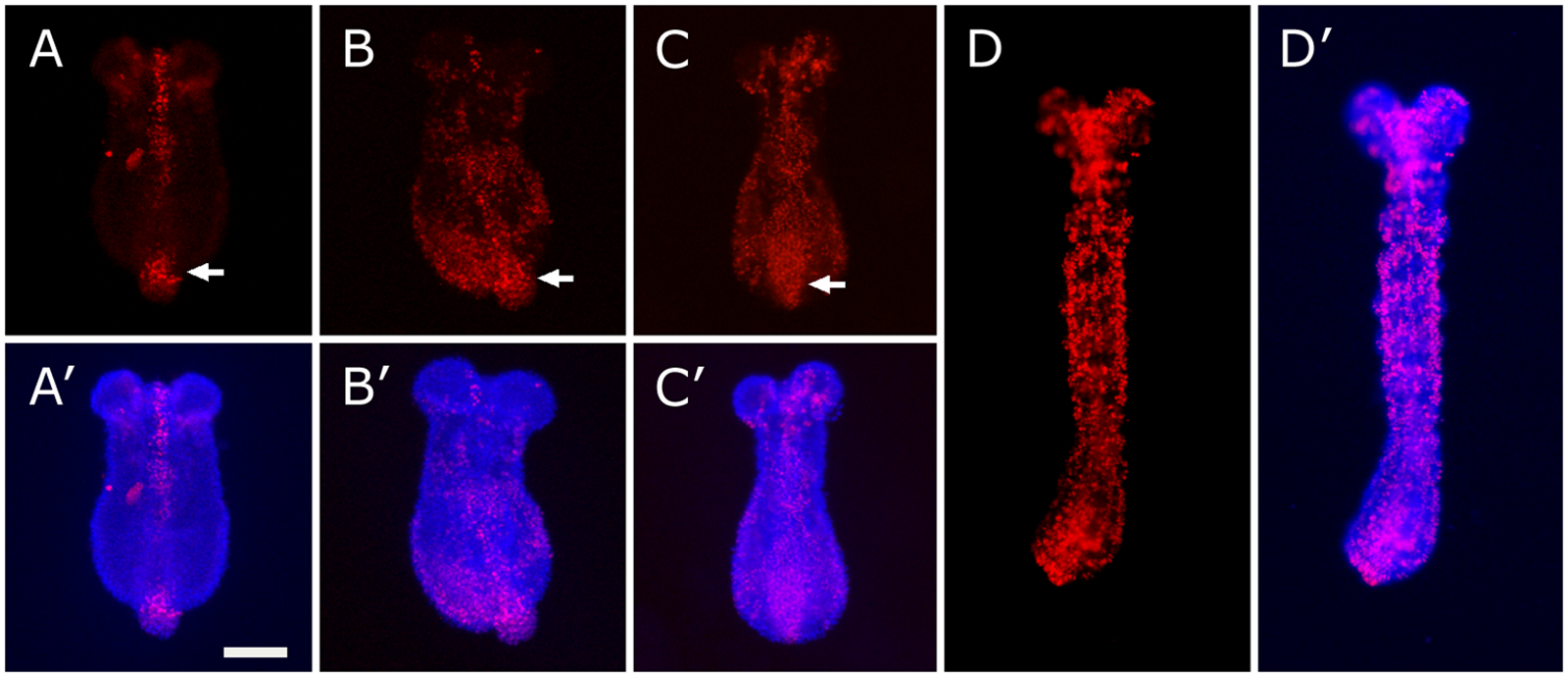
Cell proliferation during germband elongation evidenced by EdU incorporation. Detection of EdU-labeled cells during early- (A-C’) and mid- (D-D’) germband elongation by fluorescence microscopy. Representative pictures of embryos exposed to EdU during 30 minutes (A, A’), 60 minutes (B, B’D, D’) and 90 minutes (C, C’) (*n* = 12 for each time exposure). (A-D) EdU labeling in red. (A’-D’) Dapi (blue) and EdU (pink) merged images. Arrows point to the high degree of proliferation at the caudal end. All embryos are dorsally oriented. Anterior is to the top. Scale bar: 100 μm.

The generation of all posterior segments from the SAZ, a relatively small region at the end of the embryo, prompted us to think that cell proliferation must be important to give rise to a long and fully extended germband. However, cell behavior during posterior growth in *Tribolium* has been little studied until very recently and the focus has been placed mainly on cell rearrangements rather than cell proliferation [11, 12, 17, 21, 24, 26]. In order to determine the extent of contribution of cell proliferation to the posterior elongation in *T*. *castaneum* embryos, we evaluated and measured DNA synthesis *in vivo* by using the thymidine analog 5-ethynyl-2’-deoxyuridine (EdU), which is incorporated into DNA at the S-phase of the cell cycle [32]. First, we incubated dissected germbands during 30, 60 and 90 minutes with 200 μM EdU (Fig 1A–1C’), starting at the horseshoe stage (early elongation; approximately one *Tc-engrailed* stripe, see later) [17, 19]. We found a significant amount of cells proliferating after the 60- and 90-minute pulses of EdU exposure (Fig 1B–1C’), a little earlier than we previously detected using TOPRO-3 iodide [12]. This small difference probably reflects the fact that TOPRO-3 dye stains DNA in a non-cumulative way, unlike EdU, which is cumulative. We also found similar results after a 1 h EdU pulse at a later stage (Fig 1D and 1D’), *i*.*e*. during the rapid appearance of the first abdominal segments (mid-elongation; approximately 6–7 *Tc-engrailed* stripes, see later) [21]. Despite a moderate variability in the distribution and intensity of labeling among embryos (*n* = 12 for early stages and *n* = 10 for mid stages), 1 h EdU staining revealed that cell division is apparently dispersed along the elongating germband at both stages (see below), with a higher proportion of labeled cells at the caudal end (arrows in Fig 1).

### Contribution of cell proliferation to elongation is global and posteriorly biased

**Fig 2:**
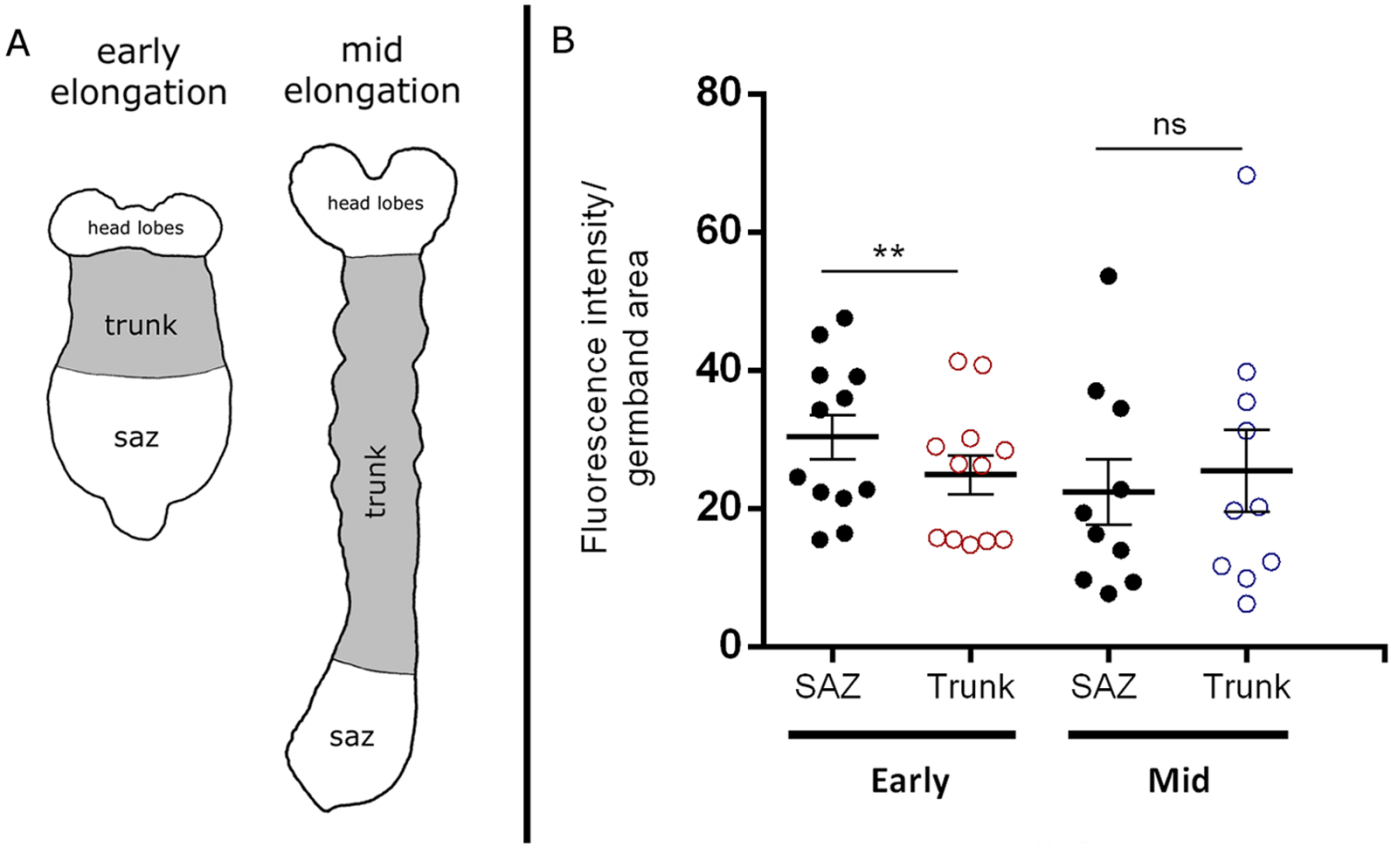
EdU fluorescence intensity comparison. (A) Schematic diagrams showing the different regions analyzed at both stages: The segment addition zone (SAZ) and the trunk region, depicted in gray in both diagrams. (B) Fluorescence intensity per germband area measurements after 1 h EdU incorporation at early- (*n* = 12) and mid-elongation stages (*n* = 10). Comparison was performed between SAZ and trunk without considering the head lobes. Error bars represent the standard error of the mean; **P <*0.001*. Ns = Non-significant.

Having established that a considerable amount of cell division takes place during early- and mid-germband extension, we wondered whether this provision of new cells comes from a localized source. Since DNA labeling intensity correlates with EdU concentration and the length of the pulse [32], we decided to quantify the relative fluorescence intensity (FI) of each embryo in order to compare cell division patterns between the SAZ, from where new abdominal segments arise progressively during germband elongation, and the trunk of the embryo, which contains the segments that were already formed (Fig 2A). When we compared EdU fluorescence intensities per area, between SAZ and trunk regions of each germband (Fig 2A), cell proliferation levels in the SAZ appeared to be greater than those observed in the trunk when it was analyzed at early-elongation stages (Fig 2B). When we analyzed themat mid-elongation stages (Fig 2A), the difference between the SAZ and trunk disappeared (Fig 2B). Moreover, we decided to compare the results obtained using fluorescence intensities with those obtained counting EdU^+^ cells within each territory (S2, S3 and S4 Figs). At early elongation, we found in average (*n* = 12), 146 EdU^+^ cells in the SAZ and 126 EdU^+^ cells in the trunk. Divided by the respective average area, we found a statistically significant increase in the amount of proliferation within the SAZ (0.8 ± 0.4) compared to the trunk (0.5 ± 0.3) (S2 and S4 Figs). We found similar results when we counted EdU^+^ cells at mid elongation (S3 and S4 Figs). The data acquired counting cells resemble hugely our results using FI, supporting the analysis performed.

Very recently, a study from Auman et al. [22] showed high levels of cell proliferation at the posterior part of the SAZ operating during germband elongation in the milkweed bug *Oncopeltus*, compared to the small amount of cell divisions found at the anterior part of this region. Since we did not perform EdU incorporation studies at the stage that they analyzed (late elongation; stage A9) [22], it is not possible to correlate their findings with our results (early- and mid-elongation). In addition, sequential segmentation starts later in *Oncopeltus* (from the first abdominal segment) than in *Tribolium* (from the mandibular segment) [24, 33], making it even more difficult to compare stages between both insects. On the other hand, it is interesting to note that in *Tribolium*, Nakamoto et al. [21] clearly demonstrated the lack of posteriorly localized proliferation. However, their conclusions were inferred from marked clones in the posterior blastoderm and not from early elongating embryos, as we show here (Fig 2B). They also found that it was possible to mathematically simulate posterior segmentation without cell division, specifically during the period of rapid segment addition, the same stage where we did not find localized proliferation (mid-elongation stage, see Fig 2B). In addition, in a recent review article [24], the same group discuss current evidence and their own unpublished data in *Tribolium* and in the crustacean *Thamnocephalus* that support the higher amount of cell proliferation that we found in the SAZ (compared to the trunk) at early-elongation stages. In conclusion, the SAZ has revealed to be very dynamic and variable along the entire process of posterior growth and elongation during *Tribolium* segmentation and most probably other arthropods [12, 21, 22, 24].

### Absence of cell division impairs axial elongation

**Fig 3:**
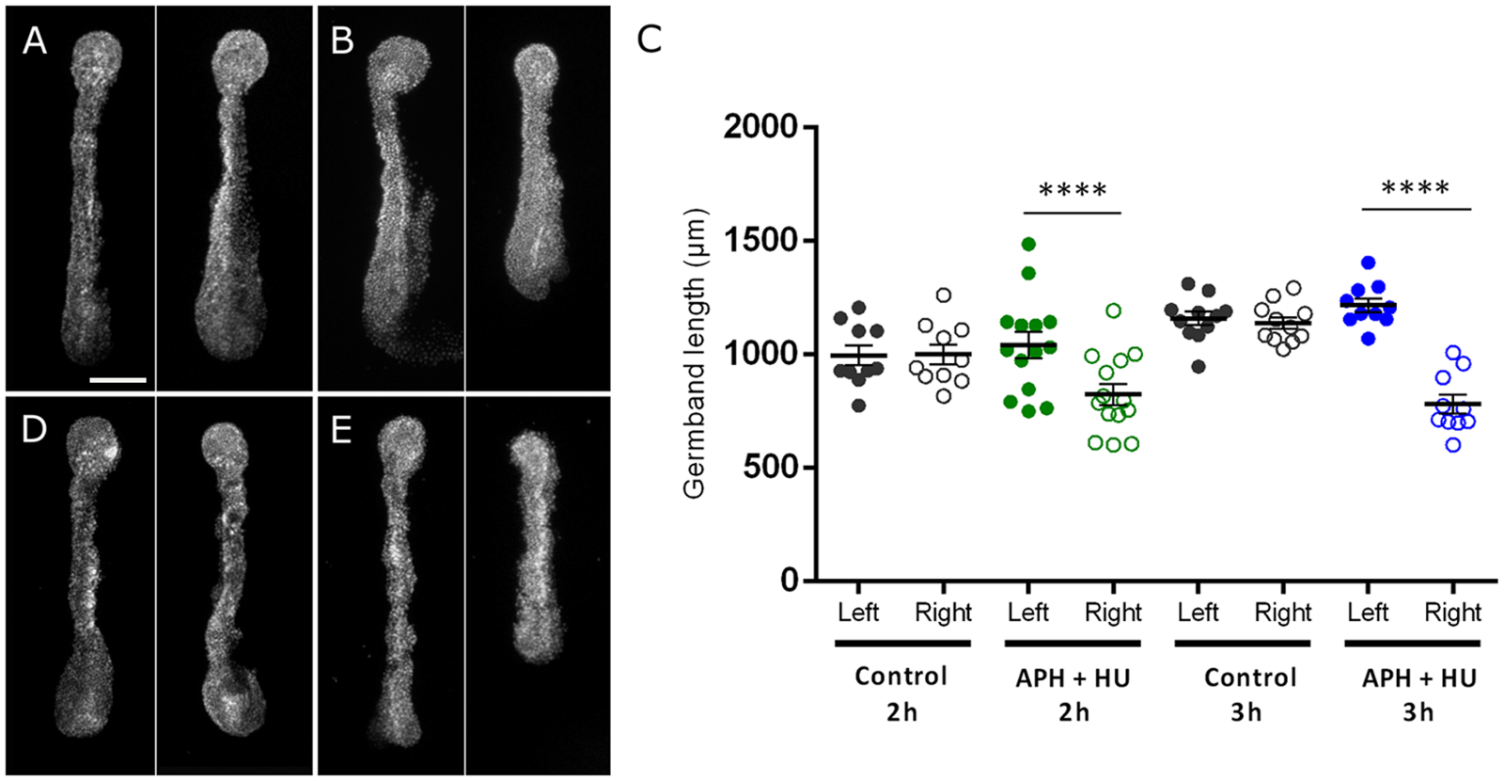
Early germband elongation in the absence of cell proliferation. A-B, D-E Representative pictures of control (left half) and treated (right half) germband halves after 2 h (A-B) and 3 h (D-E) exposition to 0.5% DMSO (sham controls; A, D) and aphidicolin+hydroxyurea (B, E). Scale bar: 100 μm. C Half-embryo length measurement after aphidicolin+hydroxyurea (APH+HU) treatment. The length in micrometers (μm) of left (control) and right (treated) halves of each bisected embryos is represented by a dot. Error bars represent the standard error of the mean; ****P <*0.00001*.

To better understand if the high levels of proliferation found during *Tribolium* elongation have a relevant role in this process, we assessed the capacity of dissected germbands to elongate when cell division is blocked by using the chemical inhibitors of DNA synthesis, aphidicolin and hydroxyurea (APH+HU) [19, 29, 30]. We used the half-embryo protocol for early elongating embryos and the pair of embryos protocol for mid-elongating embryos [see “Materials and methods” and 19]. At early stages (Fig 3), after 2 h inhibition of cell proliferation, the treated halves (right halves) were 21% shorter in length (822 ± 174 μm on average; *n* = 14) compared with the untreated control halves (left halves) (1040 ± 215 μm long on average) (Fig 3A–3C). A 3 h treatment raised the shortening effect to 36%, where proliferation-inhibited halves (right halves) showed an average length of 781 ± 131 μm (*n* = 10), whereas untreated control halves (left halves) were 1216 ± 94 μm long on average (Fig 3C–3E). A similar result was found at mid-elongation stages (S7 Fig), where dissected embryos incubated for 2 h with APH+HU were, on average, 20% shorter than untreated controls (1083 ± 111 μm vs 1352 ± 147 μm long on average, respectively; *n* = 11). Both in early- (left and right halves) and mid-elongating (couple of embryos) germbands, sham treatment controls were not significantly different (Fig 3A, 3C and 3D and S7 Fig, respectively).

**Fig 4:**
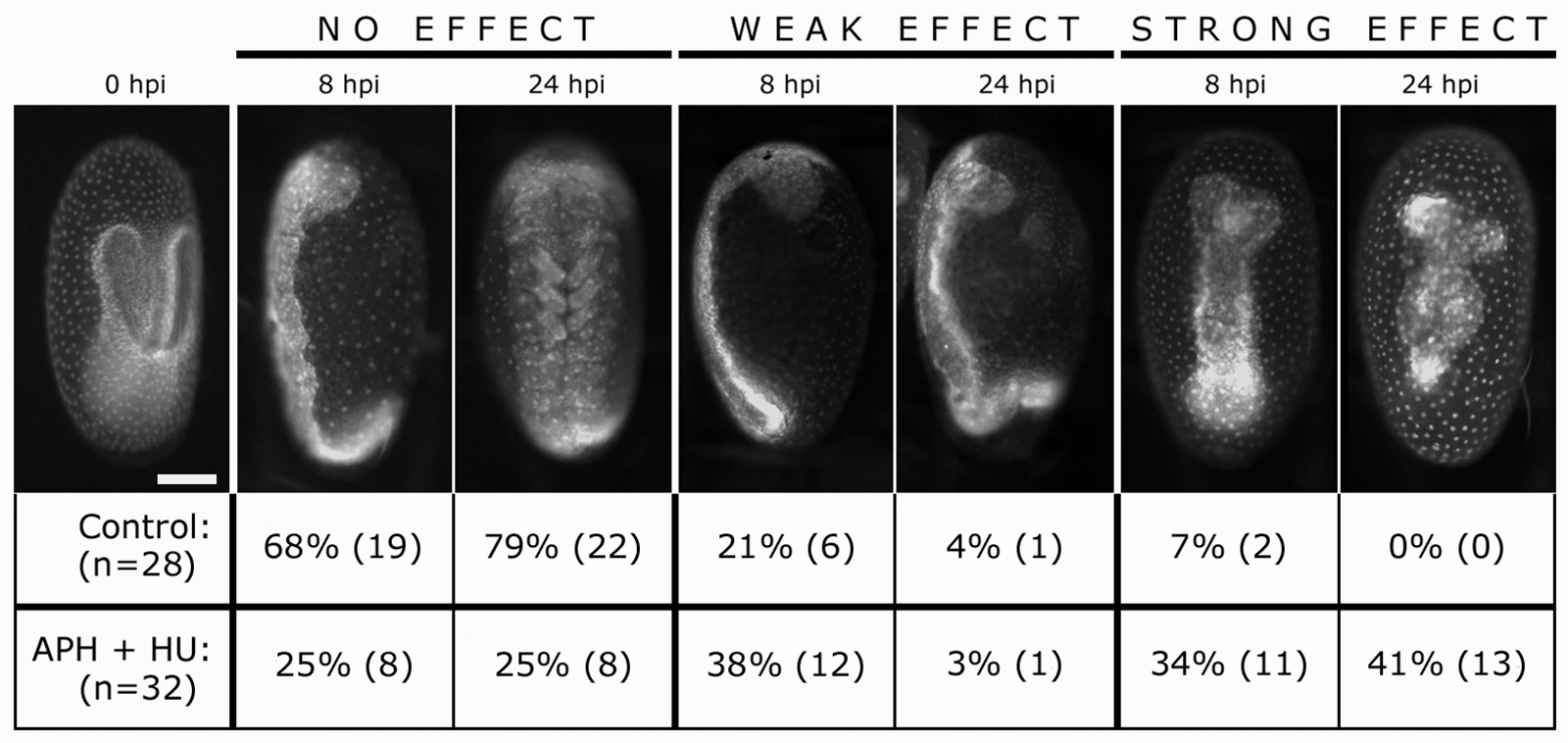
Impaired axial elongation after pharmacological injection. Early elongated embryos at the horseshoe stage (t = 0 hpi), were injected with aphidicolin+hydroxyurea, analyzed and photographed after 8 hpi and 24 hpi. Representative pictures of injected embryos are shown. The same embryo is showed at 8 hpi and 24 hpi. The percentage of the phenotypes with no effect, weak effect and strong effect are showed. Total number of control embryos is displayed in the table, as well as the number of embryos corresponding to each phenotype. The percentage of discarded embryos that were found dead at the time of analysis is not displayed. At 8 hpi, 1 embryo was discarded both in control and drug treated embryos. At 24 hpi, we discarded 5 control embryos and 10 APH+HU injected embryos because they were dead. Scale bar: 100 μm.

Our results showing that embryos unable to proliferate are less elongated, clearly demonstrate that cell division has a prominent role in axial elongation. To determine whether cell divisions are necessary to complete full germband elongation, we decided to inject APH+HU in embryos at early elongation stage. Injected embryos were analyzed and photographed after 8 and 24 hours post injection (hpi). We classified sham control and APH+HU injected embryos according to their phenotypes in No effect, Weak effect, Strong effect and Dead/discarded at both times analyzed (Fig 4). We defined a strong effect when injected embryos were evidently shorter than controls (No effect phenotype) at the same stage. In general, the effect was stronger at 24 hpi than 8 hpi, as it is shown in Fig 4. A weak effect refers to embryos with a lesser degree of shortening compared to the strong effect, possibly reflecting a slowing down of elongation. No effect corresponds to embryos that looked normally at the fluorescent stereoscope. At 8 hpi, a *Tribolium* germband with no effect spans over the complete egg length and the posterior SAZ bends around the posterior pole [34]. At 24 hpi, normal germbands already started retraction and leg buds are recognizable. Based on this classification, we found that 72% (n = 23/32) of APH+HU injected embryos were affected on their elongation when they were analyzed at 8 hpi (Fig 4), where 34% showed a strong effect, reflected in truncated germbands and 41% showed a variable degree of length shortening (weak effect), compared with 8 hpi controls (no effect embryos). On the other hand, sham control injected embryos showed only a 28% of effect, with only 2 embryos (7%) showing a strong effect. When we analyzed the same APH+HU injected embryos at 24 hpi, the percentage of strongly affected embryos raised to 41% (13/32), even considering that 31% of the embryos were discarded because they die. The fact that sham control embryos showed a high percentage of no effect (68% at 8 hpi and 79% at 24 hpi) suggests that our findings are not caused by the fact that we injected advanced embryos. In addition, analyzing embryos at two different times after injection (8 and 24 hpi) allowed us to recognize that in general, truncated embryos at 24 hpi had had a shortening effect at 8 hpi (see Fig 4). In conclusion, our findings suggest that cell division is one of the driving forces of axial elongation. Moreover, our results were similar to previous studies in vertebrates, where early elongating zebrafish embryos treated with APH+HU were shorter than controls and phenocopied the *tiy121* mutant that ceases to proliferate at the beginning of gastrulation. In addition, this mutant shows that cell division is not required for anterior-posterior patterning or the segmentation clock, but it is necessary for somite morphogenesis (similar to our findings, see below) [30]. Likewise, Williams and Nagy [24] have reported unpublished experiments in which they blocked cell division using hydroxyurea in a branchiopod crustacean. This resulted in the disruption of segment formation [24], demonstrating the important role that cell proliferation has in posterior growth, axis elongation and segment patterning in different organisms and therefore, this cellular process must be considered when interpreting genetic functions on segmentation and posterior growth.

To ensure that our treatment was indeed blocking cell proliferation, we exposed APH+HU-treated embryos to EdU. We found no EdU staining in drug-treated germbands (S1E–S1F’ Fig). In addition, to test whether the shortening effect observed after APH+HU treatments, both at early- and mid-elongation stages, was not the product of cell damage or embryo inviability, we exposed treated and untreated living germbands to the necrotic cell marker, propidium iodide (PI) [19]. Treated and control halves showed a low number of necrotic cells (S1A–S1D’ Fig), indicating that cell proliferation inhibition does not induce cell death in this model

### The establishment of the spatial pattern of segmentation is not dependent on cell proliferation

**Fig 5:**
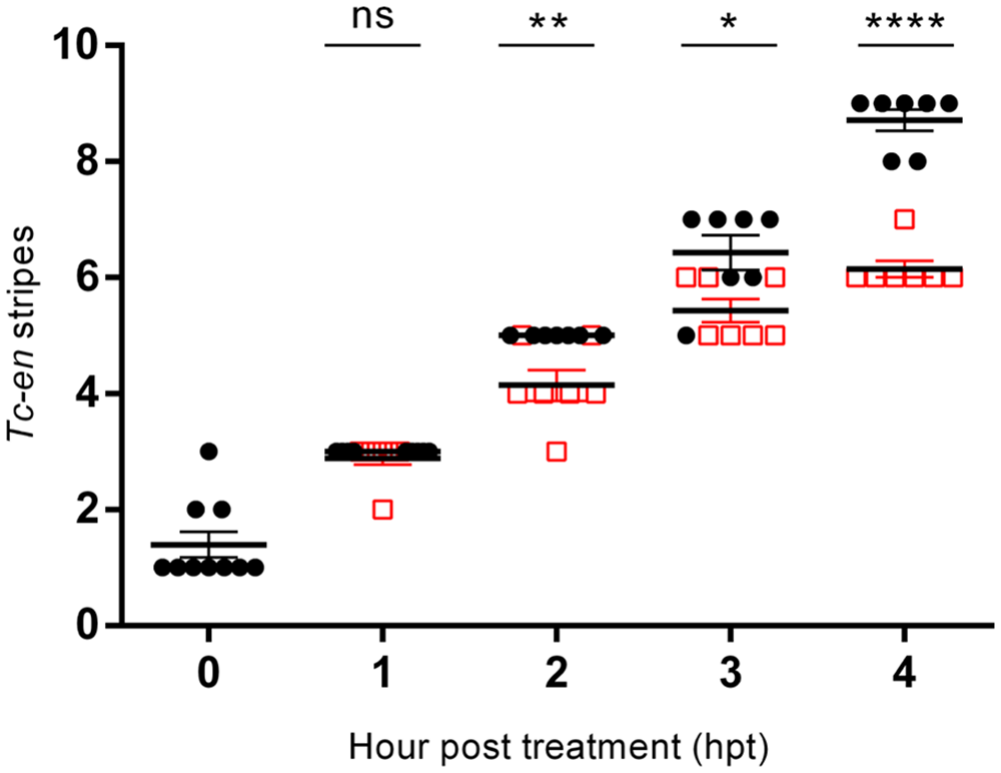
Effect of the absence of cell proliferation in the progressive formation of Tc-engrailed stripes. After blocking cell division by incubating dissected embryos at different time windows (hour post treatment), we analyzed by in situ hybridization, the formation of *Tc-engrailed* stripes. Black dots represent control embryos and red squares represent drug treated embryos. Number of embryos analyzed: 0 hpt (n = 10), 1 hpt (n = 9), 2–4 hpt (n = 7) for both, control and treated germbands. Error bars represent the standard error of the mean; Ns = Non-significant; *P <*0.01*; **P <*0.001*; ***P <*0.0001*; ****P <*0.00001*.

**Fig 6:**
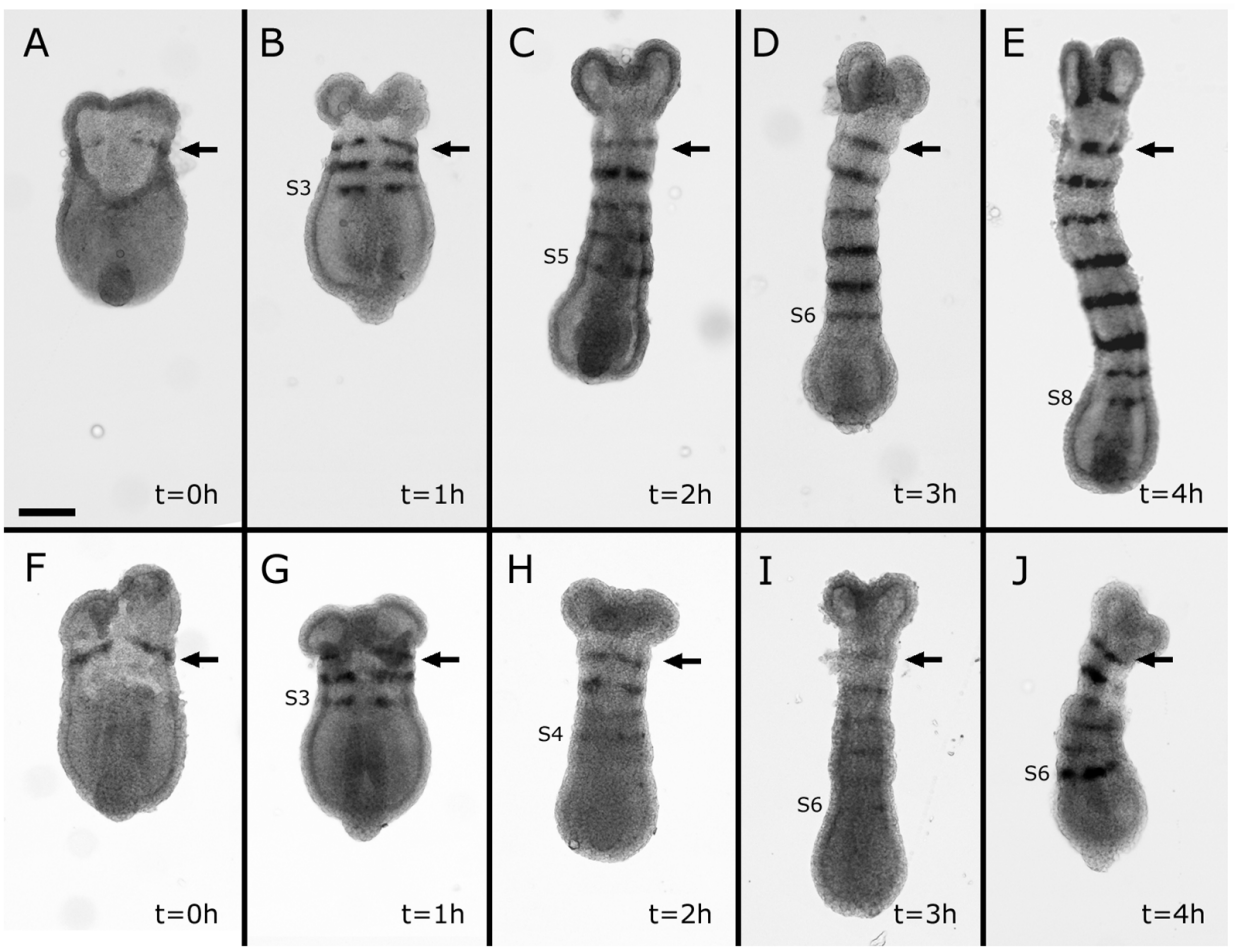
Absence of cell division does not affect the establishment of the spatial pattern of segmentation. A-J Representative pictures of embryos showing their segmental patterning by *Tc-engrailed* (*Tc-en*) *in situ* hybridization. A-E Progressive appearance of *Tc-en* stripes in control embryos at each incubation time. F-J Progressive appearance of *Tc-en* stripes in APH+HU treated embryos at each incubation time. Number of embryos analyzed: t = 0 (n = 10), t = 1 (n = 9), t = 2–4 (n = 7) for both, control and treated germbands. The black arrows point to the first *Tc-en* stripe. The last appeared *Tc-engrailed* stripe it is also indicated. All are dorsal views. Anterior is to the top. Scale bar: 100 μm.

If the shortening effect observed in APH+HU-treated embryos was caused by defects in the segmentation process, then segment patterning must be affected. To explore the role of cell proliferation in the posterior axial patterning, APH+HU-treated germbands were stained for the segmentation gene *Tc-en* by *in situ* hybridization. As we showed previously, blocking cell proliferation caused germband shortening (Fig 3); interestingly, APH+HU-treated embryos showed a progressive reduction (in average, see Fig 5 for more details) in the number of *Tc-en* stripes compared to untreated controls (Figs 5 and 6). After 1 hour of APH+HU treatment, treated germbands showed a similar number of *Tc-en* stripes than controls (3 *Tc-en* stripes in controls versus 2.9 in APH+HU embryos; Fig 5). With longer exposure to the drugs (t = 2–4 hour), whenever you see the shortening effect, those embryos inhibited to proliferate reduced the number of *Tc-en* stripes, compared to controls. For example, after 2, 3 and 4 hour treatments, control embryos showed 5, 6.4 and 8.7 stripes in average, while treated embryos showed only 4.1, 5.4 and 6.1 stripes (Fig 5). As we can see in Fig 6, the effect on the continual formation of *Tc-en* stripes is apparently without disrupting the spatial pattern of *Tc-en* expression. Nevertheless, the expression of *Tc-en* appears diffuse in the last formed segments of some embryos (Fig 6H and 6I) and the length of these segments seems to be shorter in treated embryos (see S8 Fig). Despite that blocking cell division during axial elongation leads to shorter embryos, segments formed still maintained their segmental patterning. This suggests that cell proliferation must be needed to reach the number of cells necessary to fully elongate and that this process has to be tightly coordinated with the genetic network underlying segmentation.

In conclusion, our results suggest posterior segmentation and elongation in *Tribolium* are very dynamic processes where cell proliferation contributes as an active and continuous event. We observed considerable cell division in both time windows analyzed and localized proliferation at least during early elongation. Blocking proliferation during embryo segmentation affects axis elongation, shortening the AP axis and in most severe cases causing posterior truncation. It would be interesting to explore how cell proliferation is controlled during posterior growth and which genes are involved. For example, it is well known that Wnt/β-catenin signaling is required for segmentation in *Tribolium* [35, 36], by controlling genes involved in posterior patterning, such as the transcription factor *caudal* and the pair rule gene *even-skipped* [20, 36, 37]. In this regard, it is striking that several genes required for cell division were recently found to be regulated by this signaling pathway when they were analyzed specifically in the segment addition zone of the *Tribolium* embryo [20].These results suggest that the Wnt/β-catenin pathway is most probably regulating different downstream genes in order to coordinate the establishment of the segmental patterning with cell proliferation (and other cell behaviors, like convergent extension movements), during segmentation and posterior growth.

## Supporting information

S1 Fig Effect of the absence of proliferation on cell viability and EdU incorporation. A-D’ Cell death assay using propidium iodide (PI). Representative pictures of 2 h control (left half; A, A’), 3 h control (left half; C, C’), 2 h (right half; B, B’) and 3 h (right half; D, D’) aphidicolin+hydroxyurea treated germband halves. A-D GFP (green) and PI (red) merged pictures. A’-D’ PI-labeled halves (red). The asterisk points to the posterior part of the half-embryos and “h” indicates the head lobe at the anterior part (A-D’). E-F’ Representative pictures of early elongation embryos incubated with EdU in the absence (control; E, E’) or presence of aphidicolin+hydroxyurea treatment (F, F’). Embryos marked with DAPI (E, F) and EdU (E’, F’). Note that in F’, the embryo the color of the photography was enhanced to show the red color (*n* = 6 in all the experiments). Scale bar: 100 μm.
(TIF)

S2 Fig EdU+ cells counting at early elongation stages. Table summary of the number of EdU positive cells and the areas where they were found within the SAZ and trunk, showing the statistical analysis and an example of the method using the software ImageJ. Scale bar: 100 μm.
(TIF)

S3 Fig EdU+ cells counting at mid elongation stages. Table summary of the number of EdU positive cells and the areas where they were found within the SAZ and trunk, showing the statistical analysis and an example of the method using the software ImageJ. Scale bar: 100 μm.
(TIF)

S4 Fig Edu+ cells found at early- and mid-elongation stages. EdU positive cells divided by the area of the germband analyzed (SAZ or trunk) were plotted showing differences between SAZ and trunk at both stages. Error bars represent the standard error of the mean; ****P<*0.00001*.
(TIF)

S5 Fig Determination of SAZ length by *Tc-caudal* gene expression. Representative pictures of embryos at (A) early elongation + 60 minutes and (B) mid elongation + 60 minutes expressing *Tc-caudal* gene within the entire SAZ region (black brackets). A Based on this analysis, we determined that 60 minutes after dissection, SAZ covers 37.1% of the length of the trunk (gray bracket) of the embryo (n = 5). B At mid elongation stage (+ 60 minutes), SAZ covers 25.3% of the total length of the trunk (gray bracket) of the embryo (n = 5). Anterior is to the top. All are dorsal views. Scale bar: 100 μm.
(TIF)

S6 Fig Mid elongation stage. A-B Tribolium embryo at mid elongation stage. A Representative picture of an embryo when it is selected for dissection at mid elongation stage, when the first abdominal segments start to appear rapidly according to Nakamoto et al. [21], approximately when the 6^th^– 7^th^ *Tc-engrailed* stripe arise (B), The black arrow points the first Tc-engrailed stripe and S6 shows the last formed segment marked by *Tc-en*. Anterior is to the top. A Lateral view of a GFP embryo. B Dorsal view of a flat mounted embryo. Scale bar: 100 μm.
(TIF)

S7 Fig Late germband elongation in the absence of cell proliferation. A-B Representative pictures of control (left embryo) and treated (right embryo) mid-elongated germbands after 2 h exposition to 0.5% DMSO (sham controls; A) and aphidicolin+hydroxyurea (B). Scale bar: 100 μm. C Embryo length measurement after aphidicolin+hydroxyurea treatment. The length in micrometers (μm) of each embryo is represented by a dot (control germbands) or a triangle (treated germbands). Error bars represent the standard error of the mean; ****P<*0.00001*. Ns = Non-significant.
(TIF)

S8 Fig Segment length shortening after cell division inhibition. Segment length of control and APH+HU treated embryos for incubation periods of 1, 2 and 3 hours (hour post treatment). Number of analyzed embryos: 1 hpt *n = 5* for both, control and treated embryos At 2 hpt *n* = 5 for control embryos and *n* = 6 for treated embryos. At 3 hpt, *n* = 5 for both, control and treated germbands. Error bars represent the standard error of the mean; Ns = Non-significant; * P <0.01; ** P <*0.001*.
(TIF)
