# Supplementary figures and images for: Contribution of cell proliferation to axial elongation in the red flour beetle Tribolium castaneum
Source: PLoS One. 2017 Oct 9;12(10):e0186159. doi: 10.1371/journal.pone.0186159 (PMC5633189; doi:10.1371/journal.pone.0186159)

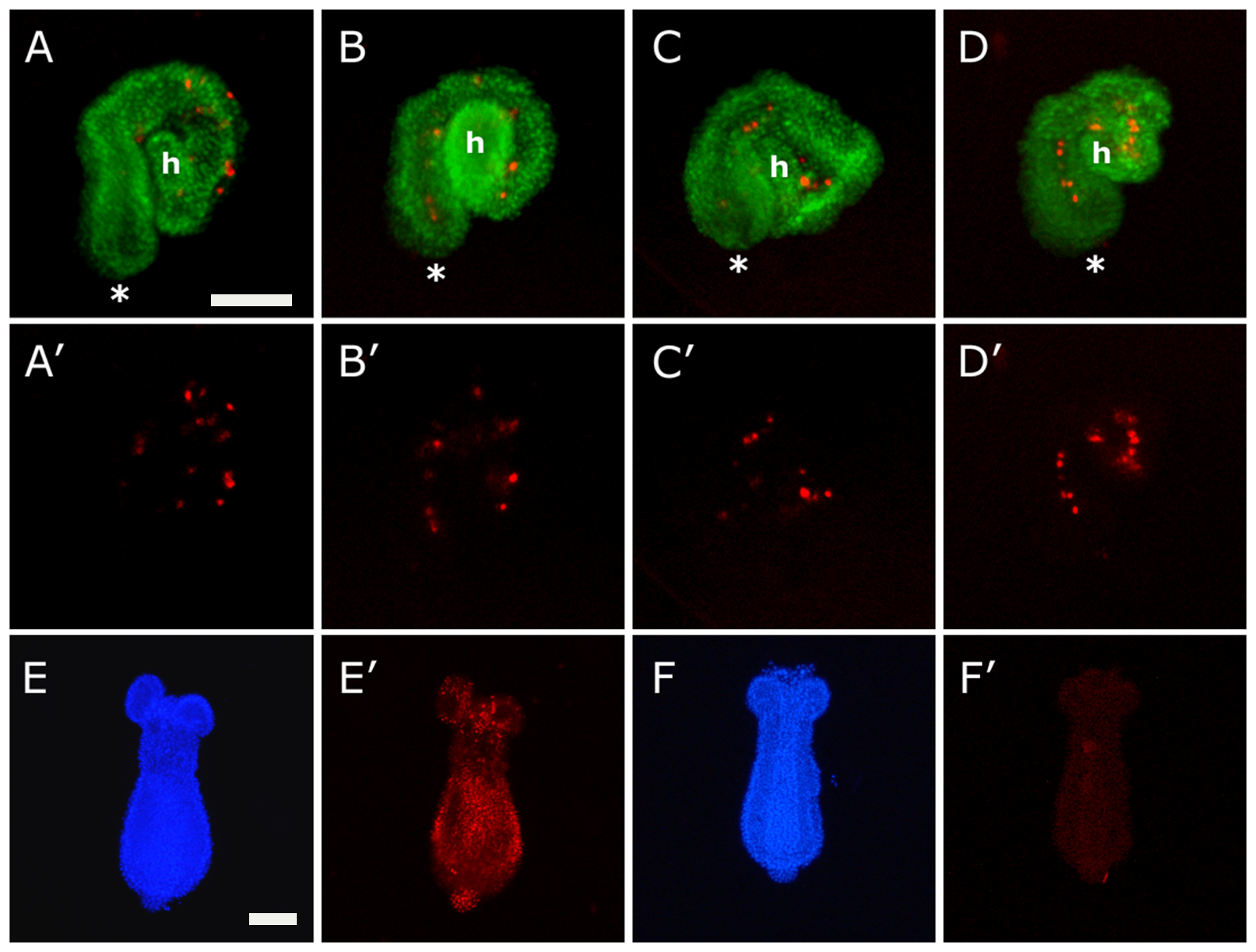

Supplement: S1 Fig — A-D’ Cell death assay using propidium iodide (PI). Representative pictures of 2 h control (left half; A, A’), 3 h control (left half; C, C’), 2 h (right half; B, B’) and 3 h (right half; D, D’) aphidicolin+hydroxyurea treated germband halves. A-D GFP (green) and PI (red) merged pictures. A’-D’ PI-labeled halves (red). The asterisk points to the posterior part of the half-embryos and “h” indicates the head lobe at the anterior part (A-D’). E-F’ Representative pictures of early elongation embryos incubated with EdU in the absence (control; E, E’) or presence of aphidicolin+hydroxyurea treatment (F, F’). Embryos marked with DAPI (E, F) and EdU (E’, F’). Note that in F’, the embryo the color of the photography was enhanced to show the red color (n = 6 in all the experiments). Scale bar: 100 μm. (TIF) [file pone.0186159.s001.tif]

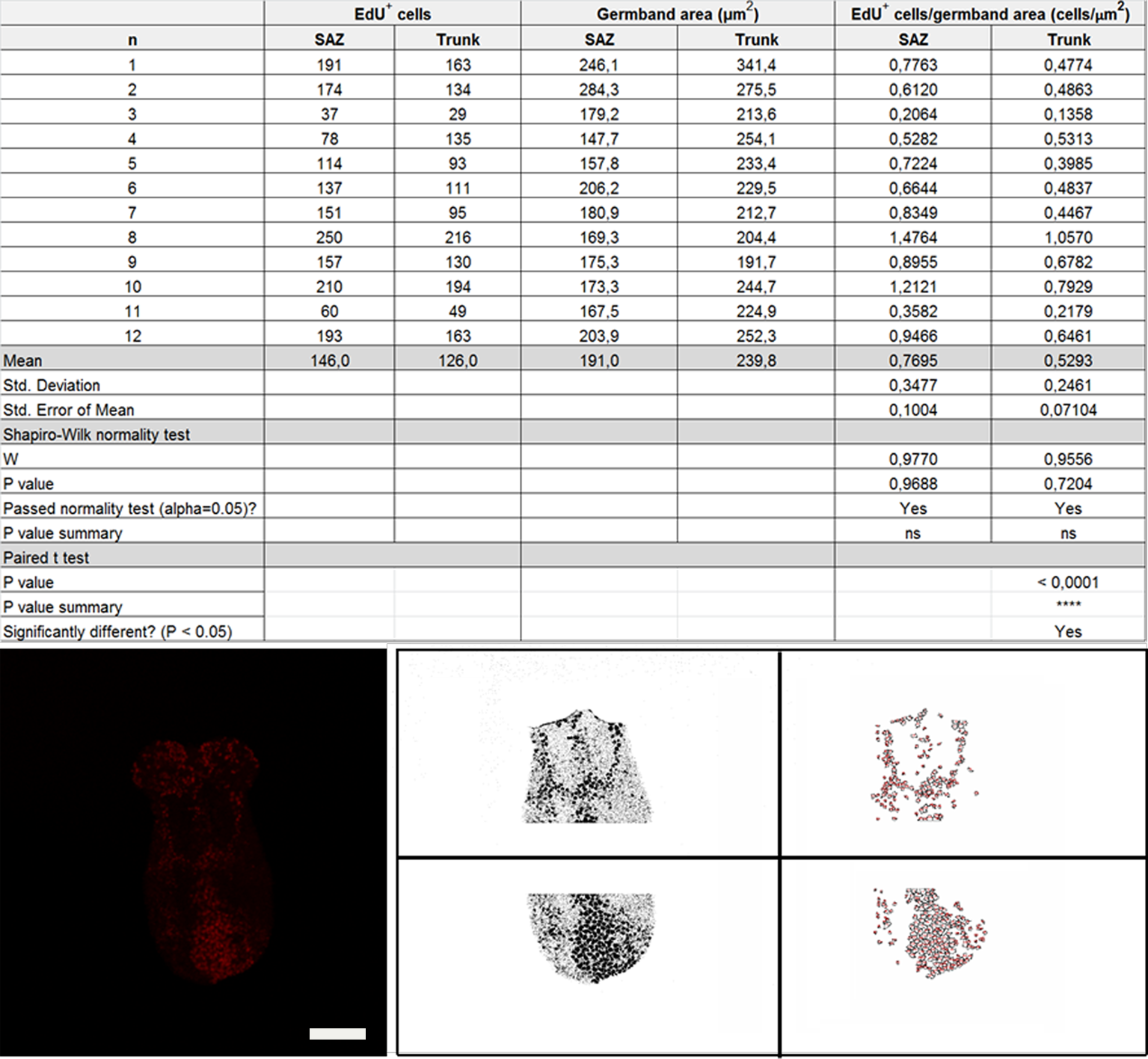

Supplement: S2 Fig — Table summary of the number of EdU positive cells and the areas where they were found within the SAZ and trunk, showing the statistical analysis and an example of the method using the software ImageJ. Scale bar: 100 μm. (TIF) [file pone.0186159.s002.tif]

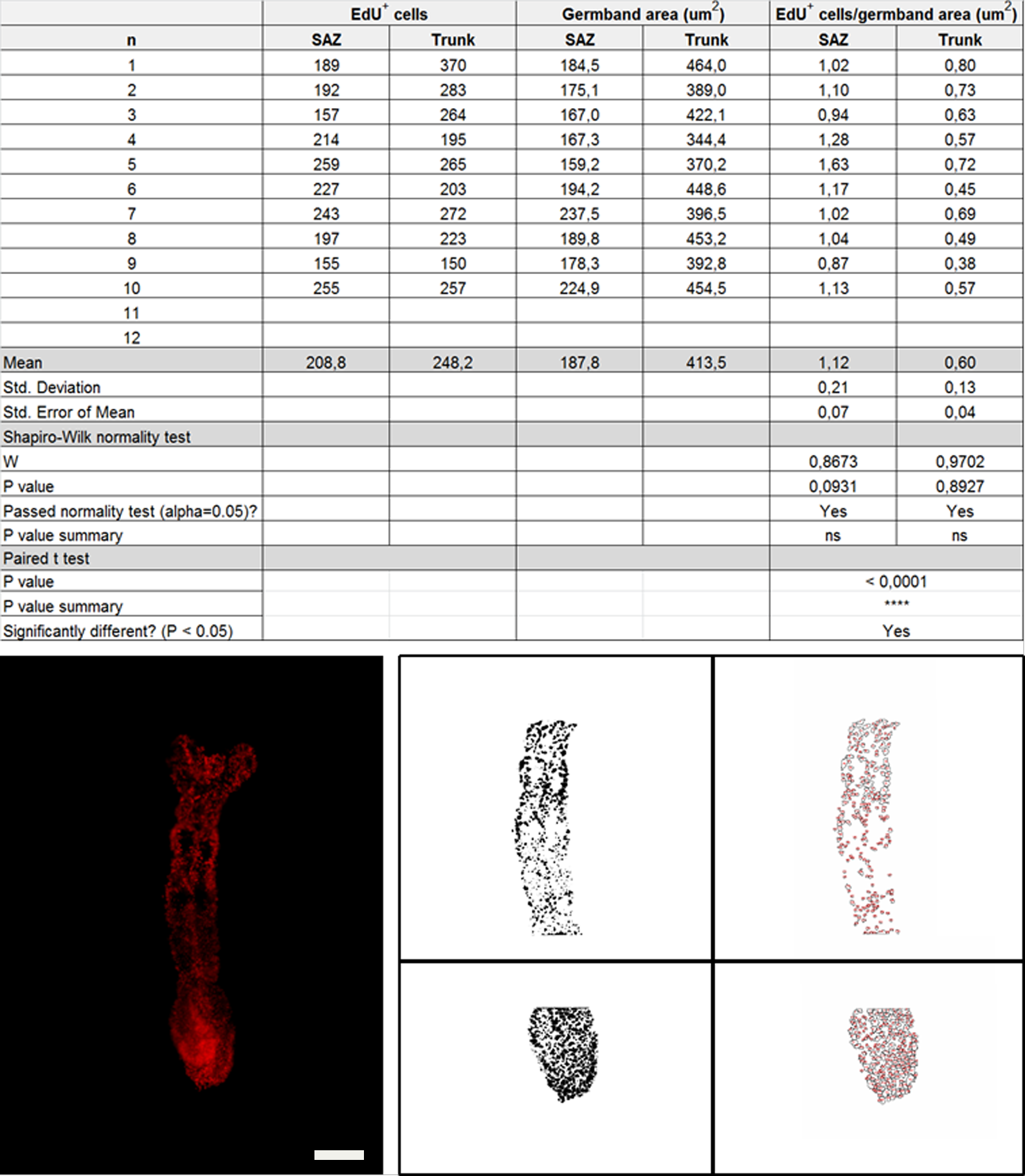

Supplement: S3 Fig — Table summary of the number of EdU positive cells and the areas where they were found within the SAZ and trunk, showing the statistical analysis and an example of the method using the software ImageJ. Scale bar: 100 μm. (TIF) [file pone.0186159.s003.tif]

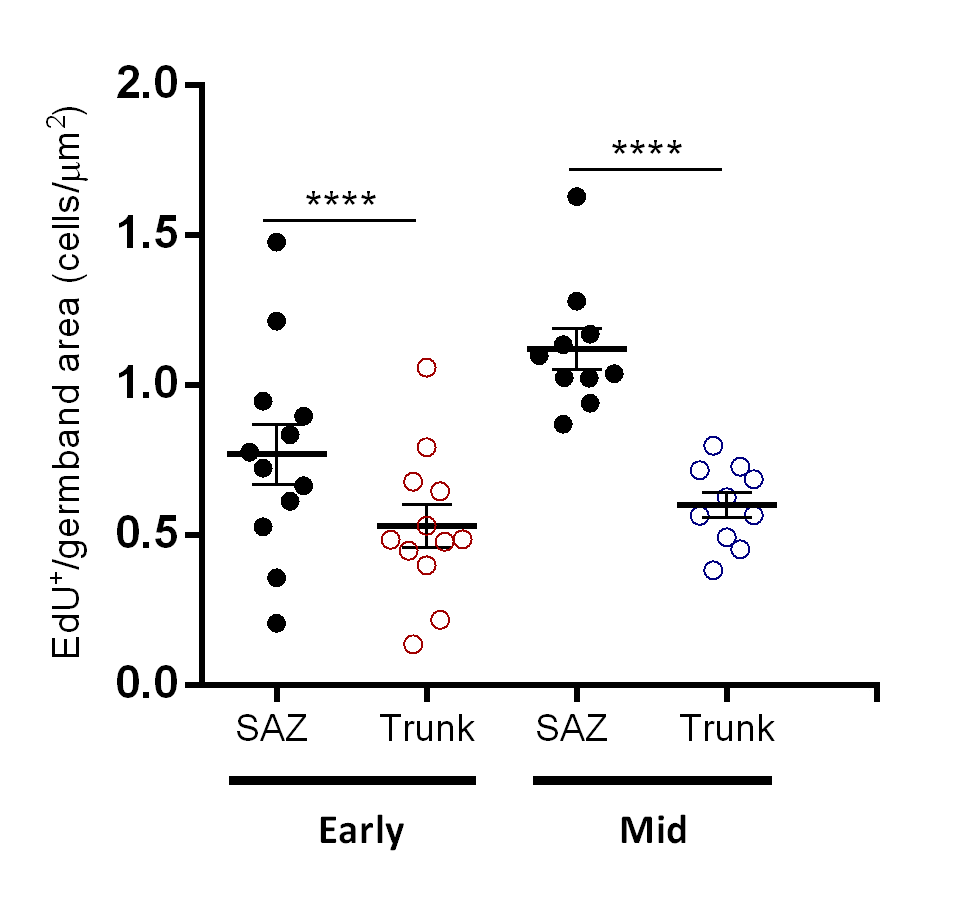

Supplement: S4 Fig — EdU positive cells divided by the area of the germband analyzed (SAZ or trunk) were plotted showing differences between SAZ and trunk at both stages. Error bars represent the standard error of the mean; ****P<0.00001. (TIF) [file pone.0186159.s004.tif]

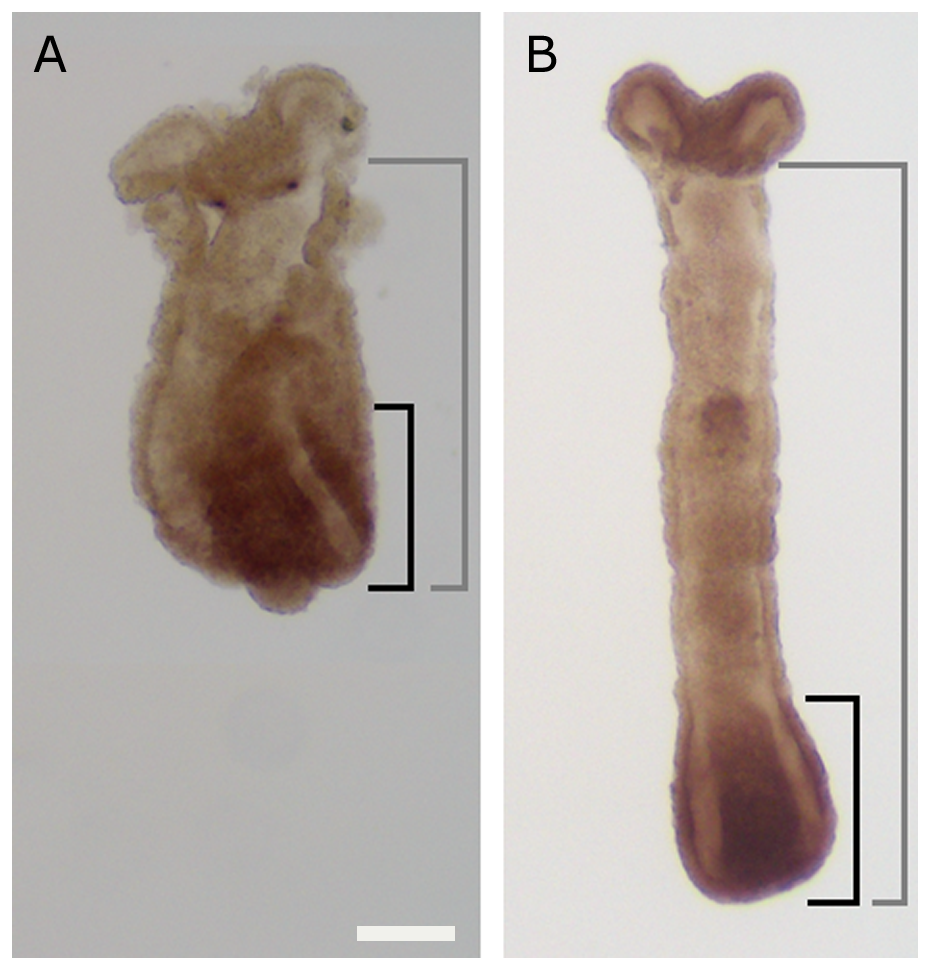

Supplement: S5 Fig — Representative pictures of embryos at (A) early elongation + 60 minutes and (B) mid elongation + 60 minutes expressing Tc-caudal gene within the entire SAZ region (black brackets). A Based on this analysis, we determined that 60 minutes after dissection, SAZ covers 37.1% of the length of the trunk (gray bracket) of the embryo (n = 5). B At mid elongation stage (+ 60 minutes), SAZ covers 25.3% of the total length of the trunk (gray bracket) of the embryo (n = 5). Anterior is to the top. All are dorsal views. Scale bar: 100 μm. (TIF) [file pone.0186159.s005.tif]

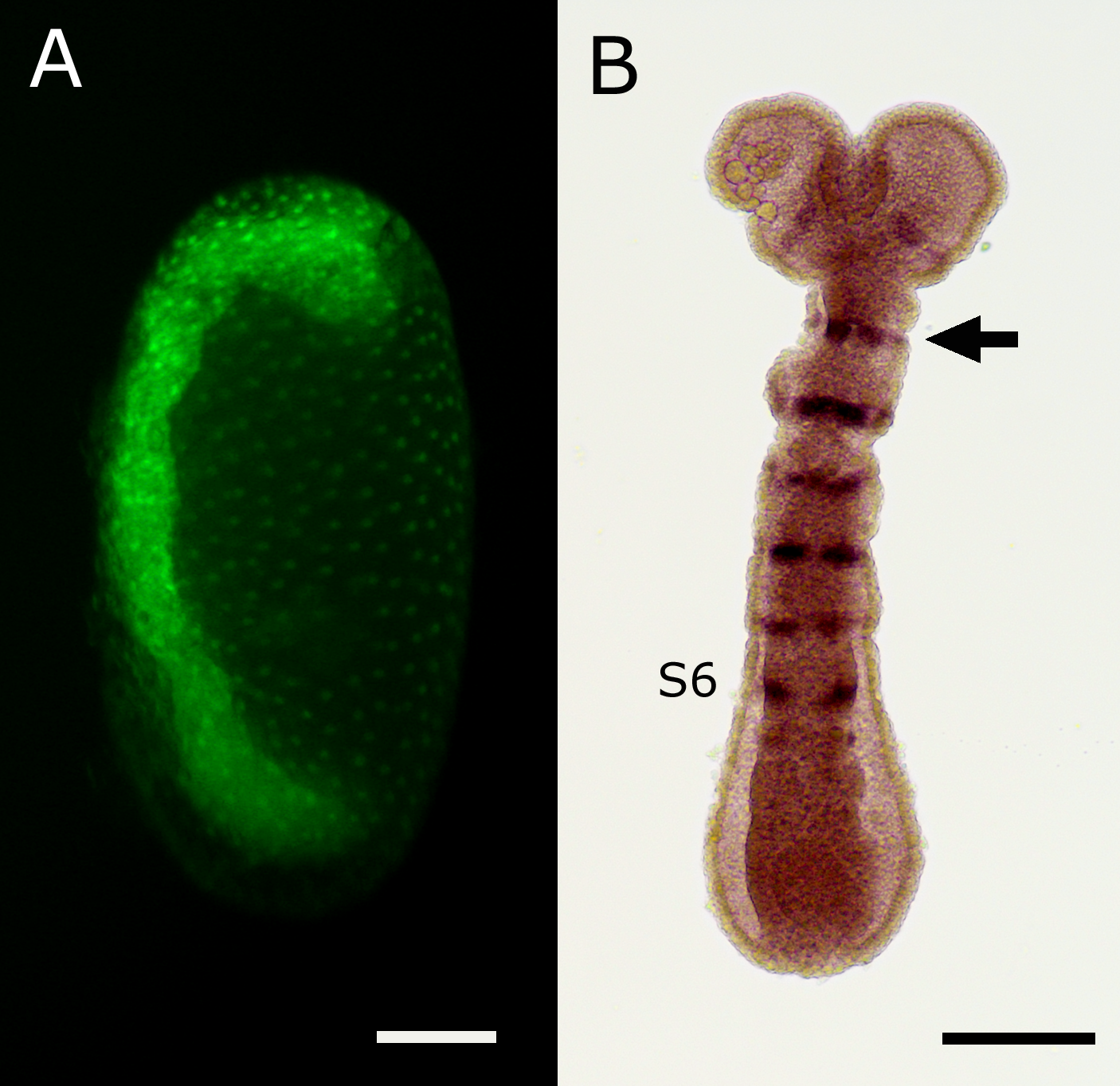

Supplement: S6 Fig — A-B Tribolium embryo at mid elongation stage. A Representative picture of an embryo when it is selected for dissection at mid elongation stage, when the first abdominal segments start to appear rapidly according to Nakamoto et al. [21], approximately when the 6th– 7th Tc-engrailed stripe arise (B), The black arrow points the first Tc-engrailed stripe and S6 shows the last formed segment marked by Tc-en. Anterior is to the top. A Lateral view of a GFP embryo. B Dorsal view of a flat mounted embryo. Scale bar: 100 μm. (TIF) [file pone.0186159.s006.tif]

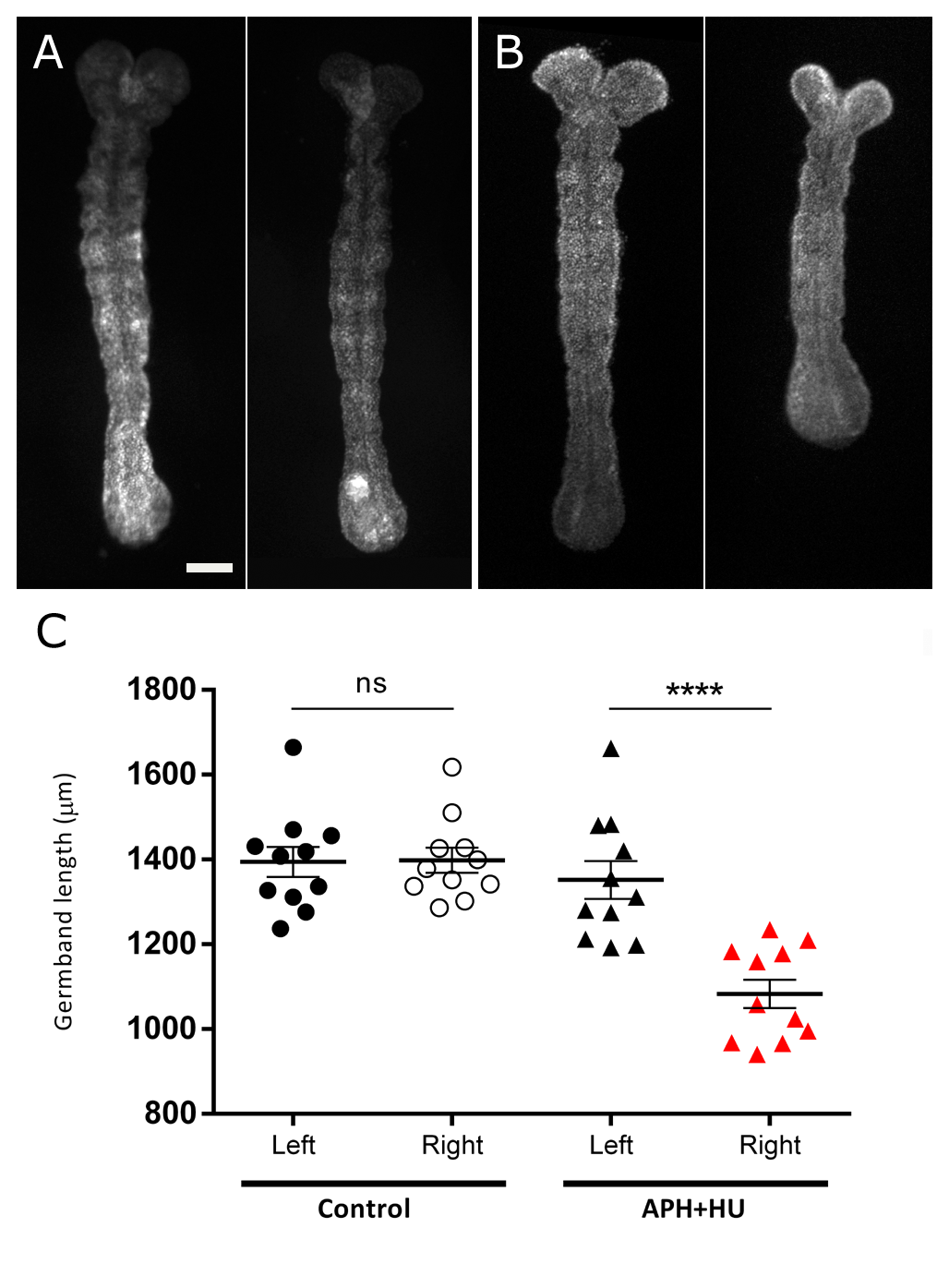

Supplement: S7 Fig — A-B Representative pictures of control (left embryo) and treated (right embryo) mid-elongated germbands after 2 h exposition to 0.5% DMSO (sham controls; A) and aphidicolin+hydroxyurea (B). Scale bar: 100 μm. C Embryo length measurement after aphidicolin+hydroxyurea treatment. The length in micrometers (μm) of each embryo is represented by a dot (control germbands) or a triangle (treated germbands). Error bars represent the standard error of the mean; ****P<0.00001. Ns = Non-significant. (TIF) [file pone.0186159.s007.tif]

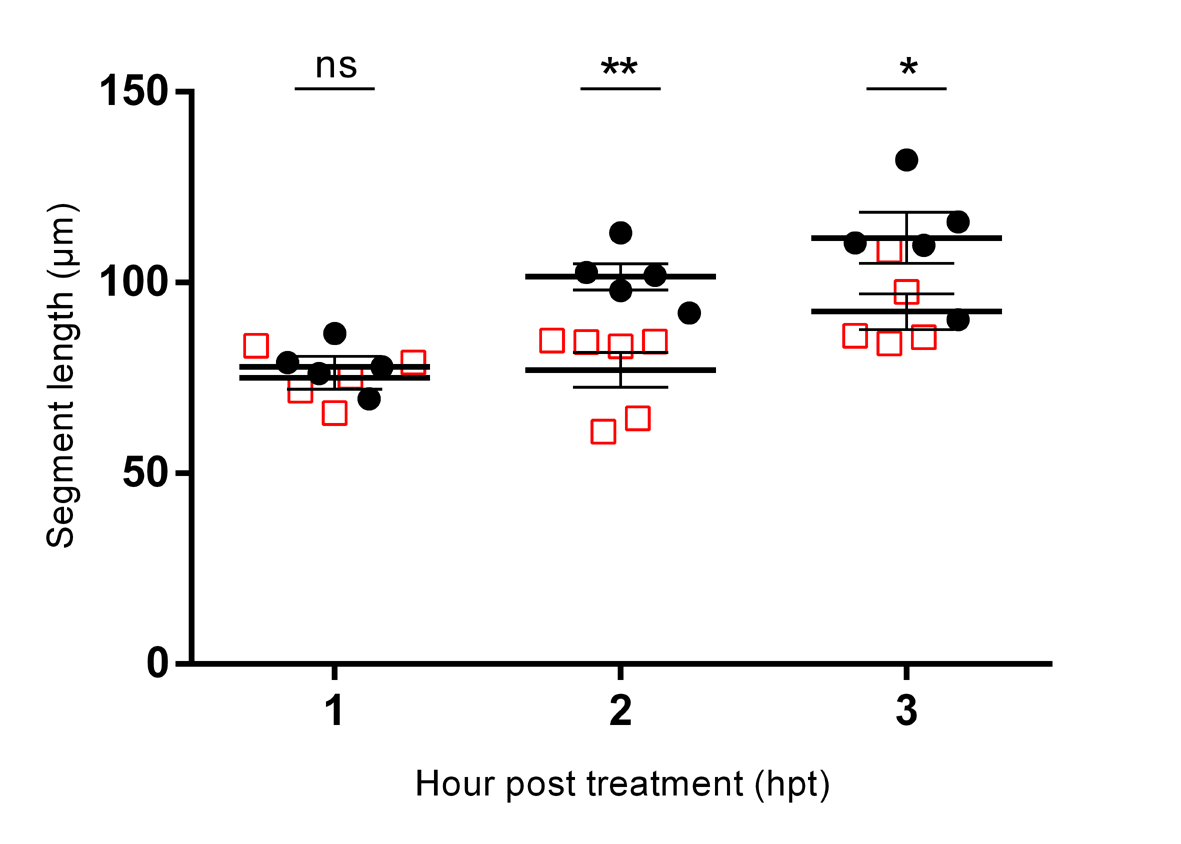

Supplement: S8 Fig — Segment length of control and APH+HU treated embryos for incubation periods of 1, 2 and 3 hours (hour post treatment). Number of analyzed embryos: 1 hpt n = 5 for both, control and treated embryos At 2 hpt n = 5 for control embryos and n = 6 for treated embryos. At 3 hpt, n = 5 for both, control and treated germbands. Error bars represent the standard error of the mean; Ns = Non-significant; * P <0.01; ** P <0.001. (TIF) [file pone.0186159.s008.tif]
